# Oleuropein Attenuates 6-Hydroxydopamine-Induced Cytotoxicity Through Redox Regulation in Differentiated Dopaminergic Neurons: Potential Involvement of RET-Associated Signalling

**DOI:** 10.3390/ijms27135892

**Published:** 2026-06-30

**Authors:** Bushra Y. Ahmed, Sigrun Lange, Mohamad Warda, Azizeh Shadidizaji, Pinar Uysal-Onganer

**Affiliations:** 1 Institute for Health & Wellbeing Research, University Square, University of Bedfordshire, Luton LU1 3JU, UK; bushra.ahmed1@beds.ac.uk; 2 Pathobiology and Extracellular Vesicles Research Group, School of Life Sciences, University of Westminster, London W1W 6UW, UK; s.lange@westminster.ac.uk; 3 Department of Physiology, Faculty of Veterinary Medicine, Atatürk University, Erzurum 25240, Türkiye; mohamad.warda@atauni.edu.tr; 4 Department of Biochemistry, Faculty of Veterinary Medicine, Cairo University, Giza 12211, Egypt; 5 Department of Agricultural Biotechnology, Faculty of Agriculture, Ataturk University, Erzurum 25240, Türkiye; 6 Coordination Office of Computer-Based Modeling and Simulation of Biological Systems, Ataturk University, Erzurum 25240, Türkiye; 7 Cancer Mechanisms and Biomarkers Research Group, School of Life Sciences, University of Westminster, London W1W 6UW, UK

**Keywords:** Parkinson’s disease, oleuropein, 6-hydroxydopamine, RET signalling, *Wnt* signalling, extracellular vesicles

## Abstract

Parkinson’s disease (PD) is a progressive neurodegenerative disorder characterized by selective loss of dopamine-containing neurons (DCNs) in the substantia nigra. Oxidative stress and impaired neurotrophic signalling contribute to PD pathogenesis. Oleuropein (Ole), a phenolic compound found in olives and olive leaves, exhibits antioxidant and cytoprotective properties that may counteract neuronal injury. This study investigated the neuroprotective effects of Ole in differentiated dopaminergic neurons (dDCNs) derived from the human ReNcell VM model exposed to 6-hydroxydopamine (6-OHDA). Ole significantly attenuated 6-OHDA-induced cytotoxicity, restoring cell viability following post- and pre-treatment compared with toxin-treated cells. Tyrosine hydroxylase expression was preserved, indicating maintenance of the dopaminergic phenotype. Ole also reduced lipid peroxidation and restored total antioxidant capacity, supporting a role in redox homeostasis. Molecular docking suggested a stable interaction between Ole and the RET receptor tyrosine kinase, suggesting a potential involvement of RET-associated survival signalling pathways that requires further experimental validation. In addition, 6-OHDA altered extracellular vesicle (EV) release and EV-associated transcripts related to *Wnt* signalling (*Wnt3a*, *Wnt5a*, GSK3), while Ole partially restored EV release profiles and EV-associated *Wnt* signalling-related transcripts. Collectively, these findings indicate that Ole protects dDCNs from oxidative stress and highlight its potential as a neuroprotective agent against dopaminergic neuronal injury.

## 1. Introduction

Parkinson’s disease (PD) is a major global health concern and represents the second most common neurodegenerative disorder worldwide. According to a World Health Organization report, approximately 8.5 million individuals were living with PD in 2019, and this number is projected to rise substantially as the global population ages [1,2]. PD is characterized by the progressive degeneration of dopaminergic neurons within the substantia nigra pars compacta, resulting in impaired dopamine signalling and the development of motor and non-motor symptoms. Although the precise mechanisms driving neuronal degeneration remain incompletely understood, extensive evidence implicates oxidative stress as a key contributor to dopaminergic neuron vulnerability. Excessive production of reactive oxygen species (ROS) can damage cellular macromolecules including lipids, proteins, and DNA, disrupt mitochondrial function, and ultimately lead to degeneration of dopaminergic neurons and disease progression [3,4,5,6,7]. Currently available treatments provide only temporary symptomatic relief and do not halt the underlying neurodegenerative process, highlighting the need for strategies that enhance neuronal resilience to oxidative stress.

Polyphenolic antioxidants have been widely investigated for their potential to counteract oxidative stress-related cellular damage [8]. In dopaminergic neurons, oxidative stress promotes lipid peroxidation due to the high polyunsaturated fatty acid content of neuronal membranes, making them particularly susceptible to oxidative injury. Lipid peroxidation generates reactive aldehydes such as malondialdehyde (MDA) and 4-hydroxynonenal, which can disrupt membrane integrity, impair mitochondrial function, and propagate neuronal damage [9,10]. Oleuropein (Ole), the principal phenolic compound found in olives and olive leaves, exhibits strong antioxidant activity, including scavenging of reactive oxygen species, inhibition of lipid peroxidation, and enhancement of endogenous antioxidant defences [11]. In addition to its antioxidant effects, Ole has been reported to exert anti-inflammatory, cardioprotective, and neuroprotective actions in several experimental models, including those relevant to neurodegenerative disorders such as PD and Alzheimer’s disease (AD) [12,13,14,15,16,17,18,19,20,21]. These findings suggest that Ole may mitigate oxidative stress-mediated neuronal injury; however, the extent to which Ole modulates lipid peroxidation and cellular antioxidant capacity in human-derived dopaminergic neurons exposed to PD-related insults remains incompletely understood.

The proto-oncogene rearranged during transfection (RET) encodes a receptor tyrosine kinase that mediates signalling by glial cell line-derived neurotrophic factor (GDNF) family ligands and plays important roles in neuronal survival, differentiation, neurite outgrowth, and synaptic plasticity [22,23]. Activation of RET signalling promotes neuronal resistance to oxidative stress through downstream pathways including phosphoinositide-3 kinase/protein kinase B (PI3K/AKT), extracellular signal-regulated kinase (ERK), and nuclear factor-κB (NF-κB), which contribute to antioxidant defence and cell survival [24,25]. Increasing evidence also indicates that oxidative stress responses intersect with additional signalling networks, including *Wnt*-associated pathways that regulate neuronal polarity, survival, and stress responses [26,27]. In particular, components of the *Wnt* pathway, such as glycogen synthase kinase-3 (GSK3) and ligands, including *Wnt*3a and *Wnt*5a, are known regulators of canonical and non-canonical signalling processes involved in neuronal stress responses [28]. Extracellular vesicles (EVs) can carry transcripts reflecting intracellular signalling activity, and analysing *Wnt* pathway-related markers within EVs may provide insight into how oxidative stress alters neuronal signalling and intercellular communication [29]. However, the extent to which antioxidant compounds such as Ole may influence these signalling mechanisms remains to be clarified.

In addition to intracellular signalling processes, EVs have emerged as important mediators of intercellular communication and potential biomarkers in neurodegenerative diseases [30]. EVs can carry proteins, lipids, and nucleic acids that reflect the physiological state of their cells of origin, and alterations in EV concentration, size distribution, and cargo composition have been reported in experimental models and clinical studies of PD [31,32,33,34,35,36,37]. These observations suggest that EV-associated molecular signatures may provide insight into disease-related cellular changes and responses to therapeutic interventions.

To address these questions, the present study investigated the neuroprotective effects of Ole in a cellular model of dopaminergic oxidative stress. Specifically, we examined whether Ole attenuates 6-hydroxydopamine (6-OHDA)-induced cytotoxicity in differentiated dopaminergic neurons, focusing on lipid peroxidation and cellular antioxidant capacity. In parallel, computational molecular docking was used to explore the potential interaction of Ole with the RET receptor as a candidate mechanism for pro-survival signalling. Finally, we examined whether oxidative stress and Ole treatment influence EV release profiles and EV-associated signalling transcripts. By integrating cellular assays, computational modelling, and EV profiling, this study aims to provide insight into the mechanisms through which Ole may support dopaminergic neuronal survival under oxidative stress conditions.

## 2. Results

### 2.1. Ole Protects dDCNs Against 6-OHDA-Induced Toxicity

**Figure 1:**
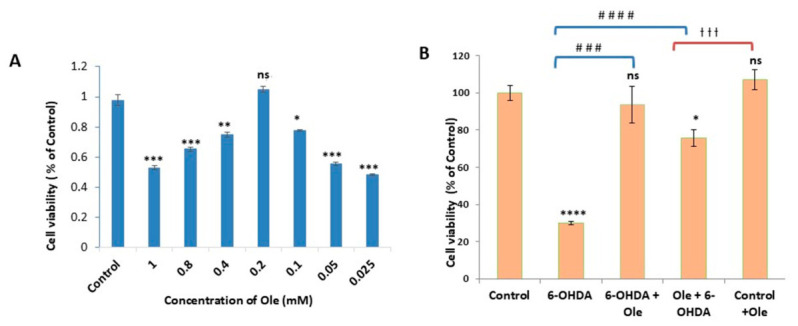
Optimization of Ole treatment and its protective effect against 6-OHDA-induced cell toxicity in dDCNs. (**A**) Dose- and time-dependent effects of Ole on dDCNs viability were assessed using the MTT assay. Cells were treated with varying concentrations of Ole (0.025–1.0 mM) for 48 h, and 0.2 mM for 24 h was selected as the optimal condition. (**B**) Cell viability was assessed by MTT assay 24 h after treatment with 0.2 mM Ole under the following conditions: Control, 6-OHDA, 6-OHDA + Ole, Ole + 6-OHDA, and Control + Ole. Viability was normalized to control (100%). Data represent mean ± SEM from three independent experiments (*n* = 3), each performed in technical triplicate. Statistical analysis was performed using one-way ANOVA followed by Dunnett’s post hoc test for comparisons versus the control group and Tukey’s multiple-comparisons test for pairwise comparisons among treatment groups. * *p* < 0.05, ** *p* < 0.01, *** *p* < 0.001, **** *p* < 0.0001 versus Control; ### *p* < 0.001, #### *p* < 0.0001 versus 6-OHDA; ††† *p* < 0.001 for the comparison between 6-OHDA + Ole and Ole + 6-OHDA. ns, not significant.

To determine the optimal Ole concentration for subsequent experiments, dDCNs were treated with increasing concentrations of Ole (0.025–1.0 mM), and cell viability was assessed after 24 h using the MTT assay (Figure 1A). Viability was normalized to untreated control cells (100%). Among the concentrations tested, 0.2 mM Ole produced the highest cell viability (107% of control) and was not significantly different from untreated controls. In contrast, both lower (0.025–0.1 mM) and higher (0.4–1.0 mM) concentrations resulted in significantly reduced cell viability. Based on these findings, 0.2 mM Ole was selected for subsequent experiments. To evaluate the protective effect of Ole against oxidative stress-induced toxicity, cell viability was assessed using the MTT assay in five experimental groups: Control, 6-OHDA, Ole, 6-OHDA + Ole, and Ole + 6-OHDA (Figure 1B). Exposure to 6-OHDA significantly reduced cell viability to approximately 30% of control levels. In contrast, Ole treatment attenuated the detrimental effects of 6-OHDA, increasing cell viability to 93.6% when administered after 6-OHDA and to 75.7% when administered before 6-OHDA. Treatment with Ole alone did not significantly affect cell viability compared with untreated controls. Collectively, these findings demonstrate that Ole protects dDCNs against 6-OHDA-induced toxicity and improves the viability of stressed cells in vitro.

**Figure 2:**
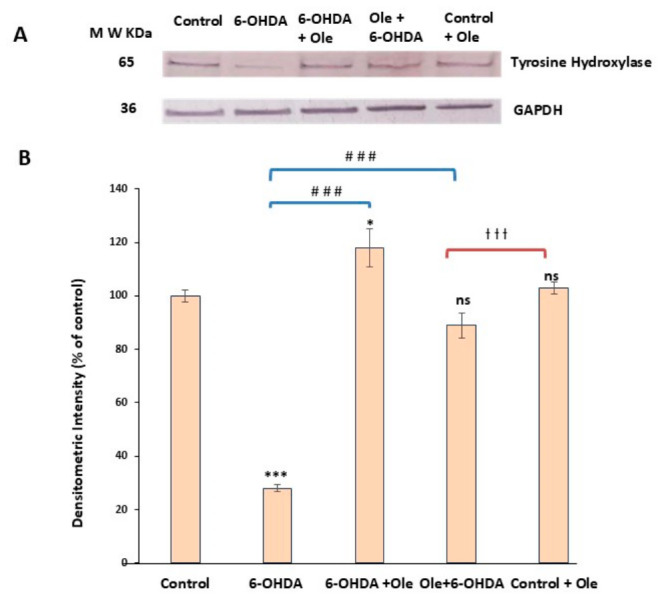
Effect of Ole on TH expression in dDCNs under 6-OHDA-induced stress. (**A**): Representative immunoblots showing GAPDH and TH expression in dDCNs across five experimental groups: Control, 6-OHDA-treated, 6-OHDA + Ole-treated, Ole + 6-OHDA-treated, and Control + Ole-treated. Cell extracts were subjected to Western blotting, and membranes were probed with antibodies against TH and GAPDH. (**B**) Densitometric values from Western blots were normalized to control (100%) and are presented as mean ± SEM from three independent experiments (*n* = 3), each performed in technical triplicate. Statistical analysis was performed using one-way ANOVA followed by Dunnett’s post hoc test for comparisons versus the control group and Tukey’s multiple-comparisons test for pairwise comparisons among treatment groups. * *p* < 0.05, *** *p* < 0.001 versus Control; ### *p* < 0.001 versus 6-OHDA; ††† *p* < 0.001 for the comparison between 6-OHDA + Ole and Ole + 6-OHDA. ns, not significant.

Tyrosine hydroxylase (TH), a marker of dopaminergic neurons was next quantified to determine whether Ole influences the maintenance of the dopaminergic phenotype under oxidative stress. Western blot analysis revealed a marked reduction in TH expression in 6-OHDA-treated cells (~28% of control). Ole treatment significantly increased TH levels relative to 6-OHDA-treated cells, maintaining expression near or above control values reaching approximately 118% and 89% of control when Ole was administered after or before 6-OHDA exposure, respectively (Figure 2), suggesting preservation of the dopaminergic phenotype under oxidative stress.

**Figure 3:**
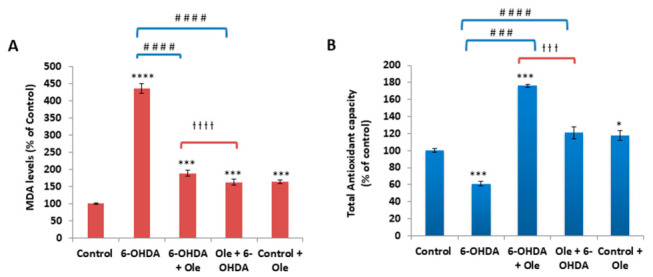
Ole attenuates 6-OHDA–induced oxidative stress in dDCNs. (**A**): Lipid peroxidation was assessed by measuring malondialdehyde (MDA) levels using a thiobarbituric acid–reactive substances (TBARS) assay in dDCNs across five experimental groups, as described in the Methods. (**B**) Total antioxidant capacity was determined using the ferric reducing antioxidant power (FRAP) assay in the same samples. MDA and FRAP values were normalized to control and expressed as a percentage of control (control = 100%). Data are presented as mean ± SEM from three independent experiments (*n* = 3), with technical triplicates averaged within each experiment. Statistical analysis was performed using one-way ANOVA followed by Dunnett’s post hoc test for comparisons versus the control group and Tukey’s multiple-comparisons test for pairwise comparisons among treatment groups. * *p* < 0.05, *** *p* < 0.001, **** *p* < 0.0001 versus Control; ### *p* < 0.001, #### *p* < 0.0001 versus 6-OHDA; ††† *p* < 0.001, †††† *p* < 0.0001 for the comparison between 6-OHDA + Ole and Ole + 6-OHDA.

To further investigate whether Ole-mediated neuroprotection is associated with modulation of oxidative stress, lipid peroxidation and total antioxidant capacity were assessed in dDCNs exposed to 6-OHDA in the presence or absence of Ole. As shown in Figure 3A, 6-OHDA treatment significantly increased MDA levels to approximately 437% of control (*p* < 0.0001), indicating pronounced lipid peroxidation. Notably, Ole treatment significantly reduced MDA levels compared to 6-OHDA alone, lowering them to approximately 189% and 163% of control when administered after or before 6-OHDA exposure, respectively. Ole treatment alone did not significantly alter MDA levels relative to control cells, indicating that Ole selectively attenuates 6-OHDA–induced lipid peroxidation without affecting basal oxidative status. Consistent with these findings, 6-OHDA significantly reduced total antioxidant capacity in dDCNs to approximately 61% of control (*p* < 0.001; Figure 3B). Notably, Ole treatment substantially restored antioxidant capacity in dDCNs compared to 6-OHDA alone, increasing levels from ~61% to 176% of control after post-treatment (6-OHDA + Ole) and to ~120% after pre-treatment (Ole + 6-OHDA) (Figure 3B): these changes represent nearly three-fold and two-fold improvements, respectively, suggesting an intrinsic antioxidant effect of Ole in dDCNs. Ole treatment in control dDCNs did not significantly alter cell viability, TH expression, or oxidative stress markers compared with control cells, indicating that Ole does not affect basal cellular homeostasis under non-stress conditions.

### 2.2. EV Profiles in Stressed dDCNs and Effects of Ole

**Figure 4:**
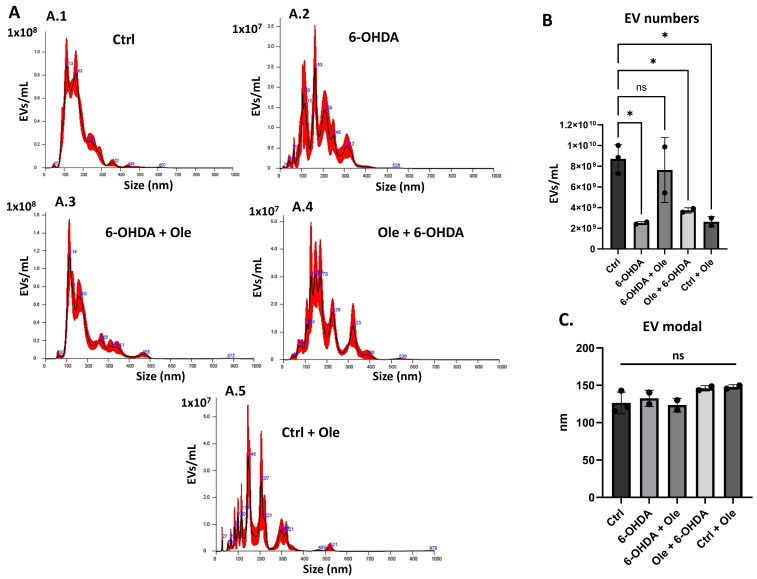
Extracellular vesicle (EV) release and size distribution following Ole and 6-OHDA treatments. (**A**) Representative nanoparticle tracking analysis (NTA) profiles of EVs isolated from control, 6-OHDA-treated, 6-OHDA + Ole-treated, Ole + 6-OHDA-treated, and control + Ole-treated dDCNs (**A.1**–**A.5**). (**B**) Quantification of EV number across treatment groups. (**C**) Comparison of EV modal size distributions across treatment groups. Data are presented as mean ± SEM from three independent experiments (*n* = 3). Statistical significance was determined using one-way ANOVA followed by Dunnett’s post hoc test versus control. * *p* < 0.05; ns, not significant.

Representative nanoparticle tracking analysis (NTA) profiles of EVs from all experimental groups are shown in Figure 4A, with size distribution in the range of mainly 40–400 nm, with slight differences observed between groups (A.1–A.5). Results revealed that 6-OHDA–treatment significantly reduced EV numbers released from dDCNs, compared with control untreated cells (Figure 4B; *p* < 0.05). Post-induction Ole treatment (6-OHDA + Ole) normalized EV release profiles bringing them closer to EV levels observed in control untreated cells (Figure 4B), albeit statistically non-significant due to individual variability, possibly due to small sample size. Pre-treatment (Ole + 6-OHDA) showed similar EV release profile as 6-OHDA only, and control dDCNs treated with Ole (Control + Ole) also showed similar reduction in EV release profiles as 6-OHDA only treatment (Figure 4B). When comparing EV modal size between the experimental groups, no significant differences were observed (Figure 4C).

### 2.3. Ole Is Associated with Changes in Wnt Signalling-Related Transcript Expression in Stressed dDCNs and EVs

**Figure 5:**
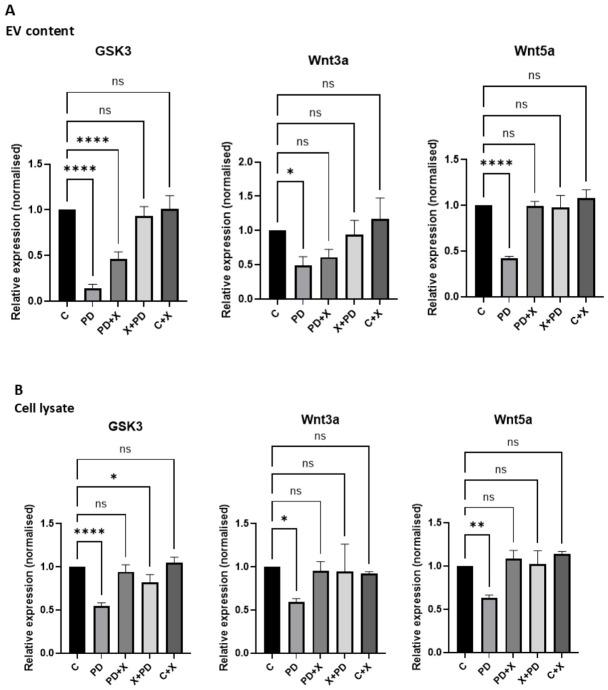
Expression of GSK3, Wnt3a, and Wnt5a in EVs and cell lysates following Ole and 6-OHDA treatment. (**A**) EV cargoes were analysed for GSK3, Wnt3a and Wnt5a. Experimental groups were the control, 6-OHDA, 6-OHDA + Ole, Ole + 6-OHDA, and Control + Ole. EV cargo analysis revealed significant reduction in GSK3, Wnt3a and Wnt5a following 6-OHDA application but was brought to more normal levels, closer to the controls, in response to Ole treatment. (**B**). Cell lysates were analysed for GSK3, Wnt3a and Wnt5a, with similar profiles as seen in EVs. GSK3, Wnt3 and Wnt5 were normalised to RPII and are presented relative to control. The Ole + 6-OHDA treated dDCNs, showing closer similarities to profiles observed in the controls. * *p* < 0.05; ** *p* ≤ 0.01; **** *p* ≤ 0.0001, ns, not significant.

To investigate whether Ole alters stress-associated signalling beyond redox regulation and RET-associated mechanisms, we quantified GSK3, *Wnt*3a, and *Wnt*5a mRNA expression levels in both dDCN cell derived EVs and in the cell lysates across all experimental conditions (Figure 5A,B).

In the EVs, 6-OHDA treatment was associated with reduced levels of *Wnt3a* and *Wnt5a* EV cargo transcripts, suggesting that resulting oxidative injury influences EV cargo composition. Post-treatment with Ole attenuated these mRNA changes, bringing them to control levels, (Figure 5A). In the cell lysates, 6-OHDA treatment was also associated with altered expression of the *Wnt* signalling-related transcripts, with a similar trend as observed for the EV cargoes, consistent with dysregulated expression of stress-response signalling-related genes (Figure 5B). Notably, Ole treatment partially normalised the expression patterns of GSK3, Wnt3a, and Wnt5a, with the strongest restorative trends observed when Ole was administered following 6-OHDA exposure (6-OHDA + Ole) (Figure 5).

### 2.4. Interactions Between Ole and RET

**Figure 6:**
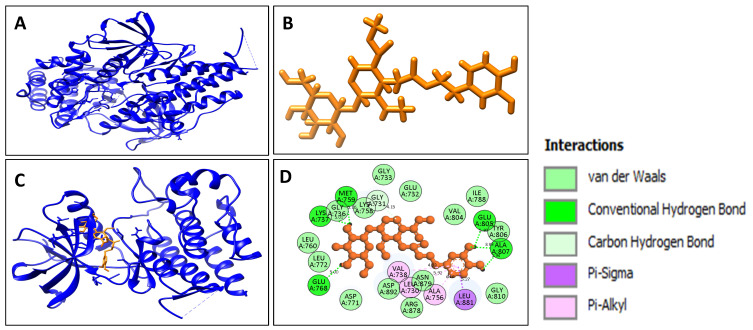
Molecular interaction of Ole with the RET receptor tyrosine kinase. (**A**) Three-dimensional structure of RET (PDB ID: 7JU6), shown as a ribbon diagram. (**B**) Chemical structure of Ole used for docking analysis. (**C**) Docked Ole–RET complex, with Ole bound to the receptor. (**D**) Two-dimensional interaction diagram highlighting key RET residues involved in binding. Interaction types are color-coded: conventional hydrogen bonds (magenta), carbon–hydrogen bonds (light green), van der Waals interactions (light green spheres), and unfavourable donor–donor interactions (red). This binding mode illustrates the critical residues stabilizing the Ole–RET complex.

To investigate the potential molecular basis of Ole’s effects, molecular docking studies were performed to characterize its interaction with the RET receptor tyrosine kinase. The three-dimensional structures of RET intracellular kinase domain (PDB ID: 7JU6) and Ole were used for docking analysis (Figure 6A,B). The top-ranked binding pose and interaction map are presented in Figure 6C, and detailed interaction parameters are summarized in Table 1, Table 2 and Table 3. The binding pocket of RET was identified, revealing a cavity with a surface area of 880.75 Å^2^, box dimensions of 22.5 × 14.5 × 16.5 Å, and a box centre located at coordinates (13.75, 0.75, −7.25), corresponding to an estimated volume of 1567.25 Å^3^. Re-docking of the co-crystallized RET inhibitor selpercatinib reproduced the experimental binding orientation with an RMSD of 0.0 Å between the crystallographic and predicted ligand poses, thereby validating the docking protocol. Molecular docking of oleuropein within the validated binding pocket yielded a top-ranked pose with a docking score of −8.7 kcal/mol, suggesting favourable binding affinity toward the RET kinase domain. Post-docking interaction profiling using PLIP identified multiple non-covalent interactions between Ole and RET (Table 1). Hydrophobic interactions were observed with residues Val738 and Leu881, suggesting stable accommodation of Ole within the hydrophobic region of the binding pocket. Hydrogen bond interactions were identified with Lys737, Met759, Glu805, and Ala807, whereas Lys758 formed salt bridge interactions that may further contribute to complex stabilization. Collectively, these interactions indicate a multimodal binding pattern involving hydrophobic contacts, hydrogen bonding and electrostatic interactions. These findings suggest that oleuropein may stably interact with the RET kinase domain within a functionally relevant binding region, although the biological consequences of this predicted interaction remain to be experimentally validated. However, the biological consequences of this predicted interaction require further experimental validation.

## 3. Discussion

### 3.1. Ole Protects dDCNs from 6-OHDA-Induced Cytotoxicity

The experimental findings of this study demonstrate that Ole significantly attenuates 6-OHDA-induced cytotoxicity in dDCNs. Exposure to 6-OHDA markedly reduced cell viability, whereas both pre-treatment and post-treatment with Ole improved cell survival compared with the 6-OHDA-treated group. Direct pairwise comparisons further demonstrated that post-treatment restored cell viability to a significantly greater extent than pre-treatment, reaching approximately 93.6% of control levels compared with 75.7%, respectively (Figure 1B). Similarly, Western blot analysis showed that TH expression was significantly increased following both treatment paradigms relative to 6-OHDA treatment alone, with post-treatment resulting in significantly higher TH levels (118% of control) than pre-treatment (89% of control) (Figure 2). These findings indicate that Ole can both protect against and mitigate 6-OHDA-induced cellular damage.

TH is the rate-limiting enzyme in dopamine synthesis and is widely used as a marker of dopaminergic neuronal integrity. The increase in TH levels observed in both Ole pre-treated and post-treated cells relative to 6-OHDA-treated cells suggests that Ole supports the maintenance of dopaminergic characteristics under oxidative stress conditions. Furthermore, the significantly greater restoration of TH expression observed following post-treatment suggests that Ole may not only confer protection prior to oxidative insult but may also promote recovery of dopaminergic neuronal function after damage has occurred.

#### Ole Modulates Oxidative Stress and Antioxidant Defences

Oxidative stress is a central contributor to dopaminergic neuronal vulnerability in experimental models of neurodegeneration. Consistent with this mechanism, 6-OHDA exposure significantly increased lipid peroxidation, reflected by elevated malondialdehyde (MDA) levels, and reduced total antioxidant capacity in dDCNs. These findings are consistent with previous reports demonstrating that 6-OHDA induces reactive oxygen species accumulation and disrupts endogenous antioxidant defences [38,39]. Treatment with Ole significantly reduced lipid peroxidation and partially restored antioxidant capacity. Direct pairwise comparisons revealed differences between the two treatment paradigms. Post-treatment with Ole (6-OHDA + Ole) resulted in significantly greater restoration of total antioxidant capacity, as determined by the FRAP assay, whereas pre-treatment with Ole (Ole + 6-OHDA) produced significantly lower MDA levels. These findings suggest that the relative efficacy of the two treatment paradigms depends on the specific aspect of oxidative stress being assessed, with post-treatment showing a stronger effect on antioxidant capacity and pre-treatment providing greater attenuation of lipid peroxidation. Importantly, Ole alone increased total antioxidant capacity without affecting basal lipid peroxidation, suggesting that Ole may enhance endogenous antioxidant defences without altering basal redox balance (Figure 3). This observation supports the possibility that Ole modulates cellular redox status particularly under conditions of oxidative stress. The partial attenuation of MDA levels in the presence of 6-OHDA suggests that Ole may reduce oxidative damage through antioxidant actions, although the underlying mechanisms were not directly investigated in the present study. These findings support the therapeutic potential of Ole in protecting dopaminergic neurons from oxidative stress-related neurodegeneration, relevant to models of PD. The lack of significant effects of Ole in control dDCNs (Figure 1B, Figure 2 and Figure 3B) suggests that its biological activity is largely context-dependent, becoming prominent only under conditions of oxidative or neurotoxic stress, hence showing physiological ceiling effects. This selective activity indicates that Ole does not disrupt normal cellular function but rather acts as a homeostatic regulator to buffer stress-induced dysregulation, a property that is desirable for neuroprotective agents.

Together, these results demonstrate that Ole significantly enhances antioxidant defences and mitigates oxidative damage in dDCNs following 6-OHDA exposure, thereby contributing to improved cell survival and preservation of dopaminergic phenotypes. Although both treatment paradigms were protective, direct comparisons indicated that their relative efficacy varied according to the endpoint measured. Post-treatment produced greater restoration of antioxidant capacity, whereas pre-treatment resulted in lower levels of lipid peroxidation. Nevertheless, both approaches substantially attenuated oxidative stress induced by 6-OHDA. The attenuation of lipid peroxidation and restoration of antioxidant capacity likely underlie the observed preservation of cell viability and TH expression. Polyphenolic compounds, including Ole, have previously been shown to enhance antioxidant enzyme systems and improve redox homeostasis in neuronal models [12,13,16,21]. In the present study, the combined improvements in cell viability, preservation of TH expression, and reduction of oxidative damage suggest that modulation of oxidative stress contributes importantly to the protective effects of Ole in dDCNs.

### 3.2. Effects of Oxidative Stress and Ole Treatment on Extracellular Vesicle Profiles

In addition to intracellular protective effects, this study examined changes in EV profiles following oxidative stress. EVs are increasingly recognized as mediators of intercellular communication and have been proposed as potential carriers of disease-related molecular signals, including in PD [31,32,33,34,35,36,37]. Exposure of dDCNs to 6-OHDA resulted in a reduction in EV concentration compared with control (untreated cells). While these EV release profile changes were observed in the current study, future studies will need to be performed for further in-depth mechanistic effects on EV biogenesis. In parallel, analysis of EV cargo content revealed reduced levels of several Wnt-related transcripts, including GSK3, *Wnt*3a, and *Wnt*5a, following 6-OHDA exposure, with partial recovery after Ole treatment. Growing evidence indicates that *Wnt* signalling pathways play an important role in dopaminergic neuron survival, neuroinflammation, and tissue repair mechanisms associated with PD [40]. Both canonical *Wnt*/β-catenin signalling and non-canonical *Wnt* pathways have been implicated in regulating neuronal differentiation, synaptic maintenance, and cellular responses to oxidative stress [27]. Recent work has demonstrated that activation of *Wnt*/β-catenin signalling in reactive astrocytes and microglia contributes to neuroprotective and regenerative processes in Parkinsonian models [41,42]. In addition, pharmacological interventions that enhance *Wnt*/β-catenin signalling have been shown to improve dopaminergic neuron survival and functional recovery in experimental PD models, including studies demonstrating *Wnt* pathway upregulation following treatment with sacubitril/valsartan in rotenone-induced PD models [43] and enhanced neuronal protection mediated by metabolic signalling modulators in MPTP [44].

In the present study, we examined transcripts associated with both canonical and non-canonical Wnt signalling pathways to explore whether oxidative stress induced by 6-OHDA is accompanied by broader pathway-level alterations in dopaminergic neurons. GSK3 is a central regulator of the canonical *Wnt*/β-catenin pathway and has been described as a key molecular switch influencing neurodegenerative processes in Parkinson’s and AD [45]. Wnt3a is a prototypical ligand involved in canonical *Wnt* signalling, whereas *Wnt*5a is typically associated with non-canonical signalling pathways that regulate cytoskeletal organization and stress responses [46]. EVs can carry RNA transcripts that reflect intracellular signalling states, profiling these *Wnt*-related transcripts within EV fractions provides an opportunity to examine whether oxidative stress alters pathway-associated molecular signatures that may be released into the extracellular environment. The observed reduction in EV-associated *Wnt*-related transcripts following 6-OHDA exposure, together with partial normalization following Ole treatment, therefore suggests that oxidative stress may influence EV cargo composition in ways that reflect altered expression of neuronal signalling-related genes. Although the present findings do not directly demonstrate activation of *Wnt* signalling, they support the concept that EV-associated transcripts could serve as indicators of stress-induced changes in signalling-related gene expression in dopaminergic neurons. The normalization of EV profiles following Ole treatment may therefore reflect broader improvements in cellular homeostasis. However, the functional relevance of these EV-associated transcripts remains unclear, and the present findings should be interpreted cautiously. A recent study on plasma-EVs from PD patients reported enrichment for *Wnt* signalling-related genes, including *Wnt*5a [37]. Further studies examining EV protein cargo, functional signalling effects, and validation in more physiologically relevant models will be required to clarify the biological significance of these observations.

### 3.3. Molecular Docking and Potential Interaction of Ole with RET

Molecular docking analysis suggested that Ole can form a stable complex within the kinase domain of the RET receptor tyrosine kinase, a key component of glial cell line-derived neurotrophic factor (GDNF) signalling [47]. The predicted binding mode involved hydrogen-bond interactions with Lys737, Met759, Glu805, and Ala807, together with salt bridge and hydrophobic contacts, suggesting a potentially stable binding orientation within the catalytic region of RET (Figure 6C). RET signalling plays an important role in neuronal survival and dopaminergic neuron maintenance through downstream pathways such as PI3K/AKT and MAPK/ERK [22,48]. Given that PI3K/AKT signalling enhances neuronal resistance to oxidative injury, the predicted interaction between Ole and RET raises the possibility that RET-associated pathways may contribute to its antioxidant and cytoprotective effects [49].

Ole has previously been reported to influence signalling pathways associated with cellular stress responses, including PI3K/AKT, ERK, and NF-κB signalling cascades [20], which are involved in cellular responses to oxidative stress and neurotoxic insults [6]. Furthermore, previous studies using activating RET variants have demonstrated alterations in dopaminergic neuron number and responses to neurotoxins such as MPTP and 6-OHDA [7,50,51], supporting the relevance of RET-associated signalling in dopaminergic neuron biology. Consistent with these findings, RET signalling engages PI3K/AKT, MAPK, and NF-κB pathways [48,52,53], while Parkin and RET have been shown to act synergistically to sustain mitochondrial function and dopaminergic neuron integrity [53]. However, the present docking analysis provides only a structural prediction of binding potential and does not demonstrate functional modulation of RET. Therefore, experimental validation through RET phosphorylation or kinase activity assays will be necessary to determine whether Ole directly influences RET signalling. Based on the present findings, RET emerges as a candidate molecular target of Ole that warrants further investigation. To our knowledge, this is the first report suggesting a potential interaction between Ole and RET, although direct evidence of RET-specific activation remains lacking [54].

#### 3.3.1. Potential Cellular Mechanisms

Although mitochondrial function and metabolic activity were not directly assessed in this study, the observed reduction in oxidative damage and improved cell viability are consistent with effects reported for Ole in other cellular systems. Ole has been shown in previous studies to stabilize mitochondrial membrane potential and attenuate endoplasmic reticulum stress responses [55]. Additionally, the non-glycosylated form of Ole has been reported to influence protein aggregation processes, including amyloid-β fibrillization, reducing toxic oligomer formation and associated cytotoxicity [56,57]. The observation that Ole improved survival in both pre-treatment and post-treatment paradigms suggests that Ole may enhances cellular resilience to oxidative stress. However, the underlying mechanisms were not directly investigated in the present study. As reported in other cellular models, the protective efficacy of Ole may decline once oxidative injury exceeds a critical threshold [58,59]. The timing and extent of Ole exposure may therefore be important determinants of its protective potential.

#### 3.3.2. Limitations and Future Perspectives

Several limitations of the present study should be considered. First, although all experiments were performed using independent biological replicates and technical triplicates where applicable, the relatively small sample size (*n* = 3 biological replicates) may limit statistical power and contribute to variability in certain datasets, particularly those involving EV and gene expression analyses. Therefore, these findings should be interpreted cautiously and confirmed in larger studies. However, the reproducibility of the overall trends across multiple independent assays supports the reliability of the principal conclusions. Although molecular docking predicted a stable interaction between Ole and the RET receptor, functional validation of RET activation or phosphorylation was not performed. Therefore, these findings represent computational binding predictions and do not directly demonstrate RET kinase activation, inhibition, or modulation of downstream signalling pathways. Additional biochemical assays will therefore be necessary to determine whether Ole directly modulates RET signalling. Furthermore, the experimental system employed here utilizes differentiated cultures derived from the human neural progenitor cell line ReNcell VM. While this model provides a human-derived and experimentally tractable platform for investigating dopaminergic-like neuronal responses to oxidative stress, differentiated cultures may retain characteristics of developmentally immature neurons and do not fully reproduce the electrophysiological properties or cellular microenvironment of adult substantia nigra dopaminergic neurons in vivo. Consequently, the findings should be interpreted within the context of an in vitro model system. Future studies should therefore aim to verify RET-dependent signalling through phosphorylation assays, investigate mitochondrial and redox homeostasis in greater detail, further in-depth characterization of EV cargoes and mechanistic effects on EV release profiles, and evaluate Ole’s efficacy in additional physiologically relevant experimental models.

## 4. Materials and Methods

### 4.1. Laboratory-Based Analysis

#### 4.1.1. Chemicals and Antibodies

The reagents included the following: ReNVM NSC maintenance media (SCM005; Millipore, Watford, UK). The primary and secondary antibodies included the following: anti-tyrosine hydroxylase (TH) and anti-GAPDH antibodies were obtained from Millipore, Hertfordshire, UK, and Abcam, Cambridge, UK, respectively. Goat anti-rabbit IgG-HRP was used as a secondary antibody and was purchased from Millipore, Hertfordshire, UK. For detection, 3,3′,5,5′-tetramethylbenzidine (TMB) solution was purchased from Thermo Scientific, Loughborough, UK.

#### 4.1.2. Cell Culture and Treatments

ReNVM-derived dopaminergic neurons (dDCNs; Millipore, Watford, UK) were differentiated and cultured for MTT and Western blot (WB) analyses as previously described [6,7]. Oxidative stress was induced by treating dDCNs with 100 µM 6-OHDA for 2 h following established protocols [6,7]. To determine the optimal concentration and exposure time for Ole treatment, dDCNs were exposed to varying concentrations of Ole (0.05–1.2 mM) for durations ranging from 1 to 48 h. Cell viability was assessed via the MTT assay (BT30006; Cambridge Bioscience, Cambridge, UK), and 0.2 mM Ole for 24 h was identified as the optimal condition (Figure 1A).

#### Experimental Groups

The study included the following five treatment groups:

1. Control: Untreated (healthy) dDCNs that received a media change only.
2. 6-OHDA-treated: dDCNs were exposed to 100 µM 6-OHDA for 2 h, followed by media replacement and a 24 h incubation.
3. 6-OHDA + Ole-treated: dDCNs were first treated with 6-OHDA as described above, followed by incubation with 0.2 mM Ole for 24 h.
4. Ole + 6-OHDA-treated: dDCNs were pre-treated with 0.2 mM Ole for 24 h and then exposed to 6-OHDA (100 µM, 2 h), followed by media replacement and another 24 h incubation.
5. Control + Ole: dDCNs were treated with only 0.2 mM Ole for 24 h.

#### 4.1.3. MTT Assay

Cell viability was assessed via the MTT assay as previously described [7]. Briefly, dDCNs were cultured in 96-well plates to ~80% confluence. Following the respective treatments, the old media was replaced with fresh media, and 10 µL of MTT solution was added to each well. The cells were incubated at 37 °C for 4 h, and the formazan crystals were dissolved by pipetting the crystals up and down in each well. The absorbance was measured at 570 nm via a Stingray microplate reader. The absorbance values were normalized to control (100%). Statistical analysis was performed using one-way ANOVA followed by Dunnett’s post hoc test. A *p*-value < 0.05 was considered statistically significant. Three independent biological experiments were performed, each with triplicate technical replicates.

#### 4.1.4. Western Blot Analysis

Western blotting was performed on dDCNs from all five experimental groups using established protocols [6,7]. Following treatment, cells were lysed, and 50 µg of total protein was separated by 12% SDS–PAGE and transferred onto PVDF membranes. Membranes were incubated overnight at 4 °C with the primary antibody against tyrosine hydroxylase (TH; 1:2500), followed by incubation with the goat anti-rabbit HRP-conjugated secondary antibody (1:1000) for 1 h at room temperature. Detection was performed using a TMB substrate (Fisher Scientific, Loughborough, UK). Glyceraldehyde 3-phosphate dehydrogenase (GAPDH) was used as a loading control (Figure 3). Blots were scanned using a GS800 densitometer (Bio-Rad, Hertfordshire, UK), and band intensities were quantified using Quantity One software (version 4.6.8; Bio-Rad, Hercules, CA, USA). TH expression levels were normalized to GAPDH and expressed relative to control.

#### 4.1.5. Ferric Reducing Antioxidant Power (FRAP) Assay

Total antioxidant activity was measured in dDCNs from the abovementioned five experimental groups using the ferric reducing antioxidant power (FRAP) assay, according to [60]. The assay is based on the ability of antioxidants to reduce the ferric–tripyridyltriazine (Fe^3+^–TPTZ) complex to its ferrous (Fe^2+^) form, producing a blue-coloured complex. Cells from all experimental groups were lysed and centrifuged at 1500× *g* for 7 min. The resulting pellets were sonicated using an MSE probe-type ultrasonicator for 30 s at 20 kcycles/s, followed by centrifugation at 1500× *g* for 7 min. The supernatants were collected for analysis. Protein concentration was determined, and the FRAP assay was performed using 100 µg of total protein per sample to minimize variability due to differences in protein content. The FRAP reagent was freshly prepared using acetate buffer (300 mM, pH 3.6), TPTZ (10 mM in 40 mM HCl), and FeCl_3_·6H_2_O (20 mM) mixed in a 10:1:1 (*v*/*v*/*v*) ratio. Samples were incubated with FRAP reagent at 37 °C for 10 min, and absorbance was measured at 593 nm. FRAP values were normalized to the control group (set as 100%), and total antioxidant capacity was expressed as percentage of control.

#### 4.1.6. Lipid Peroxidation (MDA) Assay

Lipid peroxidation was assessed by measuring malondialdehyde (MDA) levels in the five experimental groups using a lipid peroxidation (MDA/TBARS) assay kit according to the manufacturer’s instructions (Abcam, Cambridge, UK). The assay is based on the reaction of free MDA in the samples with thiobarbituric acid (TBA) to form an MDA–TBA adduct, which is quantified colorimetrically at an optical density of 532 nm. According to the manufacturer, the assay detects MDA concentrations as low as 0.1 nmol/well. Briefly, cells from all experimental groups were harvested, lysed, and centrifuged at 13,000× *g* for 10 min. The resulting supernatants were collected for analysis. Protein concentrations were determined, and samples were normalized to ensure equal protein content prior to the MDA assay. Absorbance was measured at 532 nm using a microplate reader. MDA levels were normalized to the control group (set as 100%) and expressed as a percentage of control. All measurements were performed in triplicate.

#### 4.1.7. EV Isolation and Profiling

The isolation of EVs was performed using differential centrifugation and ultracentrifugation according to previously published studies [61,62] and adhering to the MISEV guidelines [63]. Briefly, media were collected from all aforementioned experimental groups and centrifuged at 4000× *g* for 20 min at 4 °C to remove cellular debris and aggregates. The EV-containing supernatants were then centrifuged at 100,000× *g* by ultracentrifugation for 1 h at 4 °C. The EV-enriched pellets were resuspended in 1 mL DPBS (filtered through a sterile 0.22 μm filter) and ultracentrifuged again at 100,000× *g* for 1 h at 4 °C, after which the DPBS supernatant wash was discarded and the final EV pellets were resuspended in 100 μL DPBS. For EV profiling, 10 µL of EV solution was added to 990 µL DPBS for assessment by NTA.

EV concentration and size distribution profiles were assessed using the NS300 NanoSight (Malvern Panalytical Ltd., Malvern, UK), equipped with a sCMOS camera and a 488 nm diode laser, with syringe speed set at 50, camera level for capture set at 13, gain set at 1.0; while for post-processing, the detection threshold was set at 3. Four 60 s videos were collected per sample as well as averaged and replicated in histograms (representing mean and standard deviation) using the NTA software (Version 3.2.; Malvern Panalytical Ltd., Malvern, UK). EV profiles between treatments were plotted in histograms and analysed by one-way ANOVA using GraphPad Prism (version 10.6.1). EV *Wnt*-marker cargoes were assessed as described below.

#### 4.1.8. RNA Extraction and qRT-PCR

Total RNA was extracted from cell lysates and EVs from each experimental group (control, 6-OHDA, 6-OHDA + Ole, Ole + 6-OHDA, and control + Ole) as previously described [64]. RNA concentration and purity were assessed by NanoDrop spectrophotometry (ThermoFisher Scientific, Hemel Hempstead, UK) at 260 nm and 280 nm absorbance. RNA was reverse transcribed into cDNA utilising the QuantiTect reverse transcription kit (Qiagen, Manchester, UK), with the resulting cDNA acting as the template for assessing the expression of the housekeeping gene RPII, and the target genes for analysis [64]. Primer sequences are presented in Table 4. *RPII*, *Wnt3a, Wnt5a*, and *GSK3* were obtained from Integrated DNA Technologies (IDT; Leuven, Belgium). qRT-PCR was performed using the SYBR Green Master Mix (QuantiTect SYBR Green PCR Master Mix, Qiagen, Manchester, UK). The thermocycling conditions were set as follows: 95 °C for 2 min, followed by 40 cycles of 95 °C for 10 s and 56 °C for 60 s, as previously described [64,65]. The comparative 2^−ΔΔCT^ method was used to determine the relative mRNA expression levels [66], with RPII serving as the reference gene. All reactions were performed in technical triplicates across at least three independent biological replicates.

### 4.2. Molecular Docking Analysis

The crystal structure of the RET intracellular kinase domain (PDB ID: 7JU6; resolution 2.06 Å) was retrieved from the Protein Data Bank and used for molecular docking studies. This structure comprises RET residues 705–1013, encompassing the catalytic kinase domain (approximately residues 724–1016), and was selected because it represents a high-resolution X-ray structure co-crystallized with the clinically approved RET inhibitor selpercatinib, making it suitable for investigating ligand interactions within the catalytic ATP-binding region. To validate the docking protocol, the co-crystallized ligand selpercatinib was extracted from the RET kinase structure (PDB ID: 7JU6) and re-docked into the kinase active site using the same docking parameters applied to oleuropein. The predicted pose was compared with the crystallographic ligand pose, and the root-mean-square deviation (RMSD) was calculated to assess reproduction of the experimental binding mode. The resulting RMSD value of 0.0 Å demonstrated excellent reproduction of the native binding orientation and supported the reliability of the docking procedure. The molecular structure of Ole was obtained from the PubChem database (CID: 5281544). Ole was chosen due its neuroprotective potential and established antioxidant properties [8,21] (Figure 6A,B). Prior to docking simulations, the RET protein structure was preprocessed via UCSF Chimera. This included the removal of water molecules and nonstandard residues, the addition of polar hydrogens, and the assignment of Gasteiger charges. Energy minimization was conducted to resolve steric clashes and stabilize the protein structure. Ole’s 3D conformation was optimized via MMFF94 force fields to ensure accurate docking geometry.

The druggability of pockets of RET were predicted via the covityplus Server (http://www.pkumdl.cn:8000/cavityplus/#/computation (accessed on 5 February 2023)). Molecular docking simulations were performed to explore the interaction between Ole and RET. The docking procedure was carried out via PyRx-Python prescription software version 0.8 (https://pyrx.sourceforge.io/), ensuring thorough exploration of the binding poses [67]. Flexible blind docking was used. The grid box was centred at the predicted binding site (x = 15.9366; y = 0.6165; z = 3.3331; size box x = 58.9072403622; y = 49.0385817337; z = 53.9220713806) Multiple docking runs were performed to sample diverse conformations. The best-scoring binding pose (POS1) had a binding affinity of −8.2 kcal/mol. Ole and RET interactions were then characterized via the protein–ligand interaction profiler (PLIP) online server. This provided detailed profiling of noncovalent interactions, including hydrogen bonding, hydrophobic contacts, salt bridges, and π-stacking. To visualize the interactions between Ole and RET, a two-dimensional (2D) interaction diagram was generated via the Biovia Discovery StudioVisualizer v21.1.0.20298 version (Biovia 2021 [68]). This diagram highlights key residues involved in the binding process and facilitates a clear understanding of the molecular interactions at the active sites (Figure 6C).

### 4.3. Statistical Analysis

Data are presented as mean ± SEM from at least three independent biological experiments, each performed with technical triplicates where applicable. Statistical analyses were conducted using one-way analysis of variance (ANOVA). Dunnett’s post hoc test was used for comparisons versus the control group. For experiments in which direct comparisons between treatment groups were required, Tukey’s multiple-comparisons test was additionally performed. A *p* value < 0.05 was considered statistically significant.

## 5. Conclusions

In summary, this study demonstrates that Ole attenuates 6-OHDA-induced cytotoxicity in differentiated dopaminergic-like neurons, including through mechanisms associated with reduction of oxidative stress and enhancement of cellular antioxidant capacity. Ole treatment improved cell viability, preserved tyrosine hydroxylase expression, and reduced lipid peroxidation following neurotoxic insult. Both pre-treatment and post-treatment with Ole improved cellular outcomes following 6-OHDA exposure, although their relative efficacy varied depending on the biological endpoint assessed. Computational modelling further suggested a potential interaction between Ole and the RET receptor, identifying RET-associated signalling as a candidate pathway for further investigation, although this interaction requires experimental validation. In addition, oxidative stress altered extracellular vesicle release profiles and EV-cargo associated *Wnt*-related transcripts, with partial normalization observed following Ole treatment. Collectively, the reported observations provide new insights into the protective effects of Ole in a cellular model of dopaminergic oxidative stress and support further investigation of Ole as a candidate neuroprotective compound in experimental models relevant to neurodegenerative disorders.

## Figures and Tables

**Table 1:** Molecular docking analysis showing hydrophobic interactions formed between Ole and 7JU6. Hydrophobic contacts between the ligand and residues lining the active site are listed, including residue identity, interaction distance (Å), and atom indices participating in the ligand–protein interactions.

Index	Residue	AA	Distance	Ligand Atom	Protein Atom
1	738A	VAL	3.92	18	297
2	881A	LEU	3.48	19	1559
3	881A	LEU	3.76	23	1560

**Table 2:** Molecular docking analysis showing hydrogen bond interactions between Ole and 7JU6. Hydrogen bond interactions established between the ligand and active site residues are presented, including interacting amino acid residues, hydrogen–acceptor distance, donor–acceptor distance (Å), donor angle (°), and the corresponding donor and acceptor atoms involved in the ligand–protein complex stabilization.

Index	Residue	AA	Distance H-A	Distance D-A	Donor Angle	Donor Atom	Acceptor Atom
1	737A	LYS	2.27	2.88	117.45	279 [Nam]	32 [O3]
2	759A	MET	3.25	4.00	132.12	488 [Nam]	32 [O3]
3	759A	MET	2.63	3.19	115.81	32 [O3]	491 [O2]
4	805A	GLU	2.38	3.10	130.52	35 [O3]	925 [O2]
5	807A	ALA	2.06	2.98	157.29	36 [O3]	949 [O2]
6	807A	ALA	2.15	3.07	149.91	946 [Nam]	35 [O3]

**Table 3:** Molecular docking analysis showing salt bridge interactions formed between Ole and 7JU6. Ionic interactions contributing to ligand–protein complex stabilization are listed, including interacting amino acid residues, interaction distance (Å), ligand functional groups involved, and the corresponding ligand atoms participating in salt bridge formation.

Index	Residue	AA	Distance	Ligand Group	Ligand Atoms
1	758A	LYS	5.48	Carboxylate	27, 26
2	758A	LYS	5.23	Carboxylate	28, 27

**Table 4:** Primer sequences used for quantitative RT-PCR analysis of *Wnt* signalling-related genes and the reference gene RPII. Primer sequences are shown in the 5′–3′ direction.

Gene	Forward (5′–3′)	Reverse (5′–3′)
*Wnt3a*	F GTTGGGCCACAGTATTCCTC	R ATCCCACCAAACTCGATGTC
*Wnt5a*	F TCTCAGCCCAAGCAACAAGG	R GCCAGCATCACATCACAACAC
*GSK3*	F CCGACTAACACCACTGGAAGCT	R AGGATGGTAGCCAGAGGTGGAT
*RPII*	F GCACCACGTCCAATGACAT	R GTGCGGCTGCTTCCATAA

## Data Availability

The raw data supporting the conclusions of this article will be made available by the authors on request.

## References

[1] Feigin V.L., Vos T., Nichols E., Owolabi M.O., Carroll W.M., Dichgans M., Deuschl G., Parmar P., Brainin M., Murray C. (2020). The global burden of neurological disorders: Translating evidence into policy. Lancet Neurol..

[2] Su D., Cui Y., He C., Yin P., Bai R., Zhu J., Lam J.S., Zhang J., Yan R., Zheng X. (2025). Projections for prevalence of Parkinson’s disease and its driving factors in 195 countries and territories to 2050: Modelling study of Global Burden of Disease Study 2021. BMJ.

[3] Blesa J., Trigo-Damas I., Quiroga-Varela A., Jackson-Lewis V.R. (2015). Oxidative stress and Parkinson’s disease. Front. Neuroanat..

[4] Kim G.H., Kim J.E., Rhie S.J., Yoon S. (2015). The role of oxidative stress in neurodegenerative diseases. Exp. Neurobiol..

[5] Gong P., Deng F., Zhang W., Ji J., Liu J., Sun Y., Hu J. (2017). Tectorigenin attenuates the MPP+-induced SH-SY5Y cell damage indicating a potential beneficial role in Parkinson’s disease by oxidative stress inhibition. Exp. Ther. Med..

[6] Chaudhry Z.L., Klenja D., Janjua N., Cami-Kobeci G., Ahmed B.Y. (2020). COVID-19 and Parkinson’s disease: Shared inflammatory pathways under oxidative stress. Brain Sci..

[7] Chaudhry Z.L., Gamal M., Ferhati I., Warda M., Ahmed B.Y. (2022). ER stress in COVID-19 and Parkinson’s disease: In vitro and in silico evidences. Brain Sci..

[8] Li H., Deng N., Yang J., Zhao Y., Jin X., Cai A., Seeram N.P., Ma H., Li D., Yang H. (2025). Anti-inflammatory and antioxidant properties of oleuropein in human keratinocytes characterized by bottom-up proteomics. Front. Pharmacol..

[9] Angelova P.R., Horrocks M.H., Klenerman D., Gandhi S., Abramov A.Y., Shchepinov M.S. (2015). Lipid peroxidation is essential for α-synuclein-induced cell death. J. Neurochem..

[10] Lin X.M., Pan M.H., Sun J., Wang M., Huang Z.H., Wang G., Wang R., Gong H.B., Huang R.T., Huang F. (2023). Membrane phospholipid peroxidation promotes loss of dopaminergic neurons in psychological stress-induced Parkinson’s disease susceptibility. Aging Cell.

[11] Sarbishegi M. (2018). Antioxidant effects of olive leaf extract in prevention of Alzheimer’s disease and Parkinson’s disease. Gene Cell Tissue.

[12] Andreadou I., Iliodromitis E.K., Mikros E., Constantinou M., Agalias A., Magiatis P., Skaltsounis A.L., Kamber E., Tsantili-Kakoulidou A., Kremastinos D.T. (2006). The olive constituent oleuropein exhibits anti-ischemic, antioxidative, and hypolipidemic effects in anesthetized rabbits. J. Nutr..

[13] Yang D.P., Kong D.X., Zhang H.Y. (2007). Multiple pharmacological effects of olive oil phenols. Food Chem..

[14] Fabiani R., Sepporta M.V., Mazza T., Rosignoli P., Fuccelli R., De Bartolomeo A., Crescimanno M., Taticchi A., Esposto S., Servili M. (2011). Influence of cultivar and concentration of selected phenolic constituents on the in vitro chemopreventive potential of olive oil extracts. J. Agric. Food Chem..

[15] Qadir N.M., Ali K.A., Qader S.W. (2016). Antidiabetic effect of oleuropein from *Olea europaea* leaf against alloxan-induced type 1 diabetic rats. Braz. Arch. Biol. Technol..

[16] Grewal R., Reutzel M., Dilberger B., Hein H., Zotzel J., Marx S., Tretzel J., Sarafeddinov A., Fuchs C., Eckert G.P. (2020). Purified oleocanthal and ligstroside protect against mitochondrial dysfunction in models of early Alzheimer’s disease and brain ageing. Exp. Neurol..

[17] Nasrallah H., Aissa I., Slim C., Boujbiha M.A., Zaouali M.A., Bejaoui M., Wilke V., Ben-Jannet H., Mosbah H., Ben-Abdennebi H. (2020). Effect of oleuropein on oxidative stress, inflammation and apoptosis induced by ischemia-reperfusion injury in rat kidney. Life Sci..

[18] Zheng S., Huang K., Tong T. (2021). Efficacy and mechanisms of oleuropein in mitigating diabetes and diabetes complications. J. Agric. Food Chem..

[19] Malliou F., Andriopoulou C.E., Kofinas A., Katsogridaki A., Leondaritis G., Gonzalez F.J., Michaelidis T.M., Darsinou M., Skaltsounis L.A., Konstandi M. (2023). Oleuropein promotes neural plasticity and neuroprotection via PPARα-dependent and independent pathways. Nutrients.

[20] Shaibani Z., Rafieirad M. (2024). Protective effect of oleuropein on memory impairment and oxidative stress in streptozotocin-induced diabetes rats via modulation of NF-κB and Nrf2 pathways. J. Adv. Biomed. Sci..

[21] Basellini M.J., Granadino-Roldán J.M., Torres-Ortega P.V., Simmini G., Rubio-Martinez J., Marin S., Cappelletti G., Cascante M., Cañuelo A. (2025). Oleuropein aglycone influences alpha-synuclein aggregation and exerts neuroprotective effects in Parkinson’s disease models. Mol. Neurobiol..

[22] Kawai K., Takahashi M. (2020). Intracellular RET signaling pathways activated by GDNF. Cell Tissue Res..

[23] Mason I. (2000). The RET receptor tyrosine kinase: Activation, signaling and significance in neural development and disease. Pharm. Acta Helv..

[24] Smith M.P., Cass W.A. (2007). GDNF reduces oxidative stress in a 6-hydroxydopamine model of Parkinson’s disease. Neurosci. Lett..

[25] Li Q., Feng Y., Xue Y., Zhan X., Fu Y., Gui G., Zhou W., Richard J.P., Taga A., Li P. (2022). Edaravone activates the GDNF/RET neurotrophic signaling pathway and protects motor neurons from iPS cells. Mol. Neurodegener..

[26] Ferrara R., Auger N., Auclin E., Besse B. (2018). Clinical and translational implications of RET rearrangements in non-small cell lung cancer. J. Thorac. Oncol..

[27] Liu J., Xiao Q., Xiao J., Niu C., Li Y., Zhang X., Zhou Z., Shu G., Yin G. (2022). Wnt/β-catenin signalling: Function, biological mechanisms, and therapeutic opportunities. Signal Transduct. Target. Ther..

[28] Xue C., Chu Q., Shi Q., Zeng Y., Lu J., Li L. (2025). Wnt signaling pathways in biology and disease: Mechanisms and therapeutic advances. Signal Transduct. Target. Ther..

[29] Ripoll L., Zickler A.M., Vader P., El Andaloussi S., Verweij F.J., Van Niel G. (2026). Biology and therapeutic potential of extracellular vesicle targeting and uptake. Nat. Rev. Mol. Cell Biol..

[30] Hill A.F. (2019). Extracellular vesicles and neurodegenerative diseases. J. Neurosci..

[31] Guo M., Wang J., Zhao Y., Feng Y., Han S., Dong Q., Cui M., Tieu K. (2020). Microglial exosomes facilitate α-synuclein transmission in Parkinson’s disease. Brain.

[32] Picca A., Guerra F., Calvani R., Marini F., Biancolillo A., Landi G., Beli R., Landi F., Bernabei R., Bentivoglio A.R. (2020). Mitochondrial signatures in circulating extracellular vesicles of older adults with Parkinson’s disease: Results from the exosomes in Parkinson’s Disease (expand) study. J. Clin. Med..

[33] Sancandi M., Uysal-Onganer P., Kraev I., Mercer A., Lange S. (2020). Protein deimination signatures in plasma and plasma-EVs in the Brain Vasculature in a rat model of pre-motor Parkinson’s disease. Int. J. Mol. Sci..

[34] Gualerzi A., Picciolini S., Bedoni M., Guerini F.R., Clerici M., Agliardi C. (2024). Extracellular vesicles as biomarkers for Parkinson’s disease: How far from clinical translation?. Int. J. Mol. Sci..

[35] Camacho-Meño L., Labandeira C.M., Bravo S.B., Torres M.V., Bejr-Kasem H., Molina-Crespo A., Atienza M., Lanciego J.L., Cantero J.L., Kulisevsky J. (2025). Brain-derived extracellular vesicle proteomics reveals neuroprotection induced by the ARB candesartan in Parkinson’s disease patients. NPJ Park. Dis..

[36] Knab F., Lee J.H., Nirujogi R., Menden K., Braunger L., Logarnudi L., Riebenbauer B., Isik F.B., Rajkumar A.P., Czemmel S. (2025). Cellular and extracellular microRNA dysregulation in LRRK2-linked Parkinson’s disease. Mol. Neurobiol..

[37] Rajkumar A.P., Hye A., Tan S.F., Green H., Killick R., Nizamudeen Z., Isik F.B., Figueredo G., Ballard C., Svenningsson P. (2026). Transcriptomic analysis of plasma small extracellular vesicles identifies diagnostic biomarkers for Parkinson’s disease dementia. Park. Relat. Disord..

[38] Shao J., Liu X., Lian M., Mao Y. (2022). Citronellol prevents 6-OHDA-induced oxidative stress and apoptosis in SH-SY5Y cells. Neurotox. Res..

[39] Xu Q., Chen Y., Chen D., Reddy M.B. (2024). The Protection of EGCG protects against 6-OHDA-induced oxidative damage via PPARγ and Nrf2/HO-1 signaling. Nutr. Metab. Insights.

[40] Arenas E. (2014). Wnt signaling in midbrain dopaminergic neuron development and regenerative medicine for Parkinson’s disease. J. Mol. Cell Biol..

[41] Grasso M., Mascali C., L’Episcopo F. (2025). Reactive astrocytes and microglia: Wnt/β-catenin signaling in neuroprotection and repair in Parkinson’s disease. Int. J. Mol. Sci..

[42] Zhang P., Huang P., Dong Q., Luo J., Cui G., Guo X., Li M., Long X., Zhang H., Zheng W.V. (2025). Melatonin orchestrates mitochondrial fusion dynamics-mediated WNT/β-catenin signaling to promote dopaminergic differentiation of human iPS and nerve regeneration in a MPTP-induced mouse model of Parkinson’s disease. Cell Death Discov..

[43] Elkhial R.T., Awny M.M., El-Sayed E.K., Nofal S. (2026). The enhanced Wnt/β-catenin pathway upregulation by Sacubitril/valsartan via neprilysin inhibition compared to valsartan in the rotenone induced Parkinson’s disease model. Neuropharmacology.

[44] Feng P., Liu Z., Lv D., Hao W., Li D., Xue G., Bai B., Hölscher C. (2025). The novel GLP-1/GIP dual agonist DA3-CH in MPTP mouse model of Parkinson’s disease. Eur. J. Pharmacol..

[45] Liu Y., Zhang J., Tang L., Yang J., Hao L., Lou F., Su J. (2025). Glycogen synthase kinase-3: Master switch driving neurodegeneration in Alzheimer’s disease and Parkinson’s disease. Arch. Toxicol..

[46] Famili F., Naber B.A., Vloemans S., de Haas E.F., Tiemessen M.M., Staal F.J. (2015). Discrete roles of canonical and non-canonical Wnt signaling in hematopoiesis and lymphopoiesis. Cell Death Dis..

[47] Conway J.A., Ince S., Black S., Kramer E.R. (2020). GDNF/RET signaling in dopamine neurons in vivo. Cell Tissue Res..

[48] Mesa-Infante V., Afonso-Oramas D., Salas-Hernández J., Rodríguez-Núñez J., Barroso-Chinea P. (2022). Long-term exposure to GDNF induces dephosphorylation of Ret, AKT and ERK1/2 and is ineffective at protecting midbrain dopaminergic neurons in cellular models of in Parkinson’s disease. Mol. Cell. Neurosci..

[49] Meka D.P., Müller-Rischart A.K., Nidadavolu P., Mohammadi B., Motori E., Ponna S.K., Aboutalebi H., Bassal M., Annamneedi A., Finckh B. (2015). Parkin cooperates with GDNF/RET signaling to prevent dopaminergic neuron degeneration. J. Clin. Investig..

[50] Mijatovic J., Airavaara M., Planken A., Auvinen P., Raasmaja A., Piepponen T.P., Costantini F., Ahtee L., Saarma M. (2007). Constitutive Ret activity in knock-in multiple endocrine neoplasia type B mice induces profound elevation of brain dopamine concentration via en-hanced synthesis and increases the number of TH-positive cells in the substantia nigra. J. Neurosci..

[51] Mijatovic J., Piltonen M., Alberton P., Männistö P.T., Saarma M., Piepponen T.P. (2011). Constitutive Ret signaling is protective for dopaminergic cell bodies but not axonal terminals. Neurobiol. Aging.

[52] Kowsky S., Pöppelmeyer C., Kramer E.R., Falkenburger B.H., Kruse A., Klein R., Schulz J.B. (2007). RET signaling does not modulate MPTP toxicity but is required for regeneration of dopaminergic axon terminals. Proc. Natl. Acad. Sci. USA.

[53] Drinkut A., Tillack K., Meka D.P., Schulz J.B., Kügler S., Kramer E.R. (2016). Ret is essential for GDNF neuroprotective and neuroregenerative effects in Parkinson model. Cell Death Dis..

[54] Zhao Q., Bai Y., Li C., Yang K., Wei W., Li Z., Pan L., Li X., Zhang X. (2017). Oleuropein protects cardiomyocytes against apoptosis via activating the reperfusion injury salvage kinase pathway in vitro. Evid. Based Complement. Altern. Med..

[55] Elmazoglu Z., Ergin V., Sahin E., Kayhan H., Karasu C. (2017). Oleuropein and rutin protect against 6-OHDA neurotoxicity in PC12 cells. Interdiscip. Toxicol..

[56] Rigacci S., Guidotti V., Bucciantini M., Nichino D., Relini A., Berti A., Stefani M. (2011). Aβ(1–42) aggregates into non-toxic amyloid assemblies in the presence of the natural polyphenol oleuropein aglycone. Curr. Alzheimer Res..

[57] Leri M., Chaudhary H., Iashchishyn I.A., Pansieri J., Svedružić Ž.M., Gómez-Alcalde S., Musteikyte G., Smirnovas V., Stefani M., Bucciantini M. (2021). Natural Compound from olive oil compound inhibits S100A9 amyloid formation and cytotoxicity implications for preventing Alzheimer’s Disease. ACS Chem. Neurosci..

[58] Pasban-Aliabadi H., Esmaeili-Mahani S., Sheibani V., Abbasnejad M., Mehdizadeh A., Yaghoobi M.M. (2013). Olive leaf extract inhibition of 6-OHDA-induced apoptosis in PC12 cells apoptosis by olive (*Olea europaea* L.) leaf extract is performed by its main component oleuropein. Rejuvenation Res..

[59] Karković Marković A., Torić J., Barbarić M., Jakobušić Brala C. (2019). Hydroxytyrosol, tyrosol and derivatives and their health effects. Molecules.

[60] Benzie I.F.F., Strain J.J. (1999). Ferric reducing/antioxidant power assay: Direct measure of total antioxidant activity of biological fluids. Methods Enzymol..

[61] Kosgodage U.S., Uysal-Onganer P., MacLatchy A., Kraev I., Chatterton N.P., Nicholas A.P., Inal J.M., Lange S. (2018). Peptidylarginine deiminases post-translationally deiminate prohibitin and modulate extracellular vesicle release and microRNAs in glioblastoma multiforme. Int. J. Mol. Sci..

[62] Lange S., Inal J.M., Kraev I., Dart D.A., Uysal-Onganer P. (2024). Low magnetic field exposure alters prostate cancer cell properties. Biology.

[63] Welsh J.A., Goberdhan D.C., O’Driscoll L., Buzas E.I., Blenkiron C., Bussolati B., Cai H., Di-Vizio D., Driedonks T.A.P., Erdbrügger U. (2024). Minimal information for studies of extracellular vesicles (MISEV2023): From basic to advanced approaches. J. Extracell. Vesicles.

[64] Santos M., Bukhari K., Peker-Eyüboğlu I., Kraev I., Dart D.A., Lange S., Uysal-Onganer P. (2025). Hypoxia-driven extracellular vesicles promote pro-metastatic signaling in LNCaP cells via Wnt and EMT pathways. Biology.

[65] Alavanda C., Dirimtekin E., Mortoglou M., Arslan-Ates E., Guney A.I., Uysal-Onganer P. (2024). BRCA Mutations and MicroRNA Expression Patterns in the Peripheral Blood of Breast Cancer Patients. ACS Omega.

[66] Livak K.J., Schmittgen T.D. (2001). Analysis of relative gene expression data using real-time qPCR and the 2−ΔΔCT method. Methods.

[67] Trott O., Olson A.J. (2010). AutoDock Vina: Improving the speed and accuracy of docking with a new scoring function, efficient optimization, and multithreading. J. Comput. Chem..

[68] Sharma S., Sharma A., Gupta U. (2021). Molecular Docking studies on the Anti-fungal activity of *Allium sativum* (Garlic) against Mucormycosis (black fungus) by BIOVIA discovery studio visualizer, 21.1.0.0. Res. Sq..

